# Progression of Blood Flow Restricted Resistance Training in Older Adults at Risk of Mobility Limitations

**DOI:** 10.3389/fphys.2019.00738

**Published:** 2019-06-12

**Authors:** Summer B. Cook, Christopher J. Cleary

**Affiliations:** Department of Kinesiology, University of New Hampshire, Durham, NH, United States

**Keywords:** resistance training, progression, older adults, blood flow restriction, hypertrophy

## Abstract

Blood flow restriction (BFR) resistance training leads to increased muscle mass and strength but the progression leading to adaptations may be different as strength gains are often to a lesser magnitude than high-load (HL) training. The impact of training loads and repetitions on older adults’ muscle mass and strength following BFR or HL training was evaluated. Twenty-one older adults (67–90 years) classified as being at risk of mobility limitations were randomly assigned to HL (*n* = 11) or BFR (*n* = 10) knee extension (KE) and flexion (KF) training twice per week for 12 weeks. Strength was measured with 10-repetition maximum (10-RM) tests and isometric contractions. Cross-sectional area (CSA) of the quadriceps and hamstrings was measured. HL and BFR interventions increased 10-RM KF and isometric strength (*P* < 0.05) and hamstrings CSA increased an average of 4.8 ± 5.9% after HL and BFR training (time main effect *P* < 0.01). There were no differences between the training groups (time x group interactions *P* > 0.05). The rate of progression of KF training load and repetitions was comparable (time × group interactions of each variable *P* > 0.05). The groups averaged an increase of 0.50 ± 25 kg⋅week^-1^ and 1.8 ± 0.1.7 repetitions⋅week^-1^ of training (time main effects *P* < 0.05). The HL training group experienced greater improvements in KE 10-RM strength than the BFR group (60.7 ± 36.0% vs. 35.3 ± 25.5%; *P* = 0.03). In both groups, isometric KE strength increased 17.3 ± 18.5% (*P* = 0.001) and there were no differences between groups (*P* = 0.24). Quadriceps CSA increased (time main effect *P* < 0.01) and to similar magnitudes (time x group interaction *P* = 0.62) following HL (6.5 ± 3.1%) and BFR training (7.8 ± 8.2%). The HL group experienced accelerated progression of load when compared to BFR (0.90 ± 0.60 kg⋅week^-1^ vs. 30 ± 0.21 kg⋅week^-1^; *P* = 0.006) but was not different when expressed in relative terms. BFR training progressed at a rate of 3.6 ± 1.3 repetitions⋅week^-1^ while the HL group progressed at 2.2 ± 0.43 repetitions⋅week^-1^ (*P* = 0.003). HL training led to greater increases in KE 10-RM and it may be attributed to the greater load and/or faster rate of progression of the load throughout the 12-week training period and the specificity of the testing modality. Incorporating systematic load progression throughout BFR training periods should be employed to lead to maximal strength gains.

## Introduction

Skeletal muscle adaptations of muscular hypertrophy, strength, power, and endurance can be obtained through resistance training programs that specifically alter the load, sets, repetitions, and exercise volume (Garber et al., 2011). Resistance training utilizing moderate to HLs of 60–80% of an individual’s one-repetition maximum (1-RM) with 2–4 sets of 8–12 repetitions has been shown to be effective for improving muscle hypertrophy, strength and power in healthy young, and older adults while muscle endurance adaptations are achieved using low-load resistance training that incorporates less than 50% 1-RM for 2–4 sets of 15–20 repetitions (Garber et al., 2011). To elicit these adaptations, the principles of specificity, progression, and overload are incorporated over time (Ciolac et al., 2010). Progression and overload can be achieved through an increase in the number of repetitions, the load used, frequency of training sessions per week, or the volume of the resistance exercises (Kraemer and Ratamess, 2004). Specificity is achieved through the appropriate muscles involved, the movement pattern and the contraction type within the training program (Haff and Triplett, 2016).

It is recommended that older adults engage in moderate to HL resistance training protocols to combat sarcopenia with the intention to preserve physical function (Aagaard et al., 2010; Peterson et al., 2010) however, the high mechanical loads and stresses may be contraindicated due to a greater risk of injury and the greater prevalence of musculoskeletal disorders in older adults (Blyth and Noguchi, 2017). Therefore, it is important to explore alternate modalities other than HL resistance training for older adults to engage in to maximize muscle size, strength, power, and endurance.

Blood flow restricted (BFR) exercise incorporates the use of lighter loads and more repetitions than HL training. BFR exercise intervention studies or clinical trials typically last 6–12 weeks and focus on maximizing adaptations of increased muscle mass and strength (Lixandrão et al., 2018). Loads used in this exercise training are approximately 20–30% of 1-RM and participants perform multiple sets of 15–30 repetitions (Loenneke et al., 2012b; Pope et al., 2013). BFR exercise does result in improved muscle mass and strength in young adults (Laurentino et al., 2012; Martín-Hernández et al., 2013; Ellefsen et al., 2015) and older adults (Takarada et al., 2000; Karabulut et al., 2010; Vechin et al., 2015) and the adaptations have been shown to be comparable to, but not greater than the adaptations seen from HL training in both younger and older adults (Loenneke et al., 2012b; Martín-Hernández et al., 2013; Vechin et al., 2015; Cook et al., 2017). The proof of concept that BFR resistance training is an effective exercise modality has been supported. Since it may not be as effective as HL resistance training, researchers should focus on altering variables, such as cuff types, restriction pressures, loads, sets, repetitions, and volume to maximize adaptations. Considerable research has been done on BFR cuff type and restriction pressure leading researchers to summarize that restriction pressure should be relative to the cuff width and individualized to limb circumference (Mattocks et al., 2018). While studies have summarized the literature on load, repetitions, and volume (Scott et al., 2015) there are no guidelines or recommendations on the progression of BFR exercise throughout the course of a training period.

BFR exercise is an attractive clinical exercise modality for young and older adults due to the reduced stress placed on the musculoskeletal system, thus allowing individuals with mobility limitations and those with disorders or injuries to participate (Segal et al., 2015; Bryk et al., 2016; Hughes et al., 2017; Ladlow et al., 2018). Our previous research demonstrated the effectiveness of BFR resistance exercise in older adults with mobility limitations (Cook et al., 2017) and this secondary research study investigates the progression of the participants’ training loads, repetitions and volume during the 12-weeks of HL and BFR resistance training. It was speculated that the rate of change in loads and repetitions over a training period would vary between HL and BFR training which could describe the consistent findings that strength gains after BFR training are rarely greater than HL strength gains.

## Materials and Methods

### Experimental Design

A between groups repeated measures design was used to assess exercise loads and repetitions, muscle strength and CSA of older adults before and after 12-weeks of a resistance training exercise intervention. A stratified randomization approach was used to place participants by age (65–75 years and 75+ years) and sex into one of two resistance exercise interventions: HL or BFR and an attention control condition. The data for the control group is not reported in this study but can be found in a previous publication (Cook et al., 2017).

### Subject Recruitment and Participant Descriptions

Community dwelling males and females ≥65 years old were recruited to participate in this study. The recruitment approach and inclusion criteria are described in detail elsewhere (Cook et al., 2017). Briefly, older adults that were classified as at risk of mobility limitations (Manini et al., 2007) and met the health and strength criteria volunteered for the study. All participants signed an informed consent approved by the University of New Hampshire Institutional Review Board to participate in the resistance training study. The volunteers gained approval from a primary care provider to participate and underwent an exercise stress test on a treadmill supervised by a cardiologist that also provided medical clearance. These individuals then underwent a familiarization session in which they were orientated to the exercise equipment, the strength testing protocol, and the general study procedures. The short physical performance battery (SPPB) was done to assess the participants’ abilities to complete chair stands, balance tests and a 4-m walk. The SPPB scores range from 0 to 12; with 12 indicating the highest degree of lower extremity function. Participants were then randomly assigned to the HL, BFR or attention control condition. Twenty-one participants (9 males and 12 females; Table 1) aged 67–92 years old fully completed the study and their data were used in the data analysis.

**Table 1:** Descriptive data presented as mean (standard deviation) of the exercise intervention groups.

	HL	BFR
N	11 (5M, 6F)	10 (4M, 6F)
Age (years)	76.3 (8.7)	76.4 (6.6)
Mass (kg)	73.3 (10.9)	75.4 (10.9)
Height (m)	1.7 (0.1)	1.7 (0.1)
Body mass index (kg⋅m^2^)	26.5 (3.0)	27.5 (3.3)
Short physical performance battery (SPPB)	10.7 (2.1)	10.2 (1.9)


*There were no differences between the groups (*P* > 0.05).*

### Exercise Intervention

The participants underwent 12-weeks of twice per week supervised resistance training using seated KE and knee flexion (KF) machines (Body Solid GCEC340, Forest Park, IL, United States). A horizontal leg press machine (Body Solid GLP-STK, Forest Park, IL, United States) was also used in the training but this data was not used in the analysis due to the infrequency of training on this equipment in BFR exercise literature. Each exercise session consisted of a warm-up of 10 repetitions at a very light weight (∼5% of 1-RM) and progressed into three sets of each exercise performed to volitional failure with 60 s of rest between sets and 3 min between exercises. Volitional failure occurred when the individual could not complete full range of motion or ceased exercise due to perceived fatigue. Ratings of perceived exertion (RPE) using a 1–10 scale were provided by participants after each set of exercise. The participants performed only one set of each exercise for the first week, two sets the second week, and three sets for the remainder of the study. The participants assigned to the HL intervention performed the lower body exercises listed above at 70% of an estimated 1-RM. The concentric and eccentric portions of the exercise movement lasted 3 s (a rate of 20 contractions per minute) and were controlled by a metronome. Exercise load was progressed by 1–2 kg when participants were able to perform more than 15 repetitions for at least two sets of exercise on a given day. Participants assigned to the BFR training group performed the KE and KF exercise at 30% of estimated 1-RM while the leg press exercise was performed at 50% of estimated 1-RM. The load of 50% was chosen due to inability to restrict blood flow to the gluteal muscles involved in this exercise. The BFR was applied to the proximal portion of the leg utilizing a narrow (6 cm × 83 cm) pneumatic tourniquet cuffs (D.E. Hokanson, Inc., Bellevue, WA, United States) that were inflated before exercise (Hokanson TD312 Calculating Cuff Inflator, Bellevue, WA, United States). The cuff was set at approximately 1.5 times brachial systolic blood pressure which equated to an average pressure of 184 ± 25 mmHg applied to the participants. Based on a previous study that predicted arterial occlusion pressure from systolic and diastolic blood pressure and thigh circumference (Loenneke et al., 2015) the BFR pressure used in our study was approximately 66% of predicted arterial occlusion pressure. This percentage is similar to other studies (Laurentino et al., 2012; Soligon et al., 2018). The BFR cuffs remained inflated during each exercise and the rest between sets. This resulted in approximately 5 min of restriction time per exercise. The cuffs were deflated for the 3-min rest periods between exercises. Exercise load was progressed by approximately 1–2 kg when participants were able to perform more than 30 repetitions for at least two sets of exercise on a given day. Load, repetitions and exercise volume (load × repetitions) were recorded daily for all participants in the HL and BFR training interventions. The average rates of progression in load, repetitions, and volume per month were calculated from the difference in weeks 1–4, 4–8, and 8–12.

### Measurements

Strength and CSA were assessed prior to the intervention and at 12-weeks of training. The testing was spread out over 4 days that included: a magnetic resonance imaging (MRI) scan to determine CSA; strength testing on the dynamometer and 10-RM testing. The post testing began 2–4 days after the last exercise training session.

#### Cross-Sectional Area

The average CSA of the quadriceps and hamstrings muscle groups on the right leg were obtained through serial axial MRI scans. CSA was obtained from the upper leg (greater trochanter to patella) using a 1.5-T Phillips Intera whole body scanner with software Release 11 (Phillips Medical Systems, Bothell, WA, United States). Ten mm-thick transaxial images (2122-ms repetition time, 10.12-mm slice-to-slice interval) were taken after a 30-min supine rest period to allow for fluid equilibration. The images were transferred to a computer for calculation of muscle CSA using the National Institutes of Health ImageJ software (Abramoff et al., 2004). A research technician was blinded to participants’ group assignments and time points of MRI scan. To calculate CSA (cm^2^) of the quadriceps and hamstrings all the images were traced from the appearance of the distal portion of the rectus femoris to the appearance of the femoral neck. The same number of slices using the anatomical landmarks was measured for each subject at each of the testing time points (∼10 ± 1 images). Measurements were performed in duplicate and the average CSA of the quadriceps and hamstrings were used in the analysis. The test-retest reliability of CSA of the quadriceps was previously determined to have an intraclass correlation coefficient (ICC) of 0.99 (Cook et al., 2010).

#### Strength

Unilateral, isometric maximum voluntary contraction (MVC) torque at 60°; of extension (0° was full extension) was assessed on the right knee extensors and flexors on an isokinetic dynamometer (HUMAC NORM, CSMI, Stoughton, MA, United States). Participants were instructed to produce as much force, as quickly as possible, for a 3-s maximal contraction following an auditory cue. Three trials were performed, and the last two contractions were averaged. The trial-to-trial ICC of the MVC was 0.99.

Bilateral 10-RM was determined using the 10-RM approach. Briefly, participants performed a light warm-up on the KE, KF, and LP machines. Load was progressively increased until participants could perform approximately 10 repetitions. 1-RM was then predicted using an equation (Haff and Triplett, 2016) to set the exercise load for the resistance training protocols (70% of 1-RM for HL training and 30% of 1-RM for BFR training). Predicted 1-RM from the 10-RM test was also used to further adjust workout loads after 6-weeks of training.

### Statistical Analyses

The sample size for this secondary analysis study was dependent on our previous study (Cook et al., 2017). Data are expressed as mean ± standard deviation. Repeated measures analysis of variance (ANOVA) procedures were used to detect differences in the dependent variables (10-RM and MVC in KE and KF, CSA in hamstrings and quadriceps, and exercise repetitions, loads and volumes) performed by the participants with respect to the within-subjects independent variable (pre and post-training) and the between subjects training factor (HL and BFR). Significant interactions and main effects were followed with appropriate *post hoc* tests, including Tukey *post hoc* tests or *t*-tests with Bonferroni adjustments. Relative changes were calculated as pre values divided by post values and expressed as a percentage. Independent *t*-tests were used to assess the relative changes between training interventions. An alpha level of 0.05 was required for statistical significance. IBM SPSS Statistics version 24.0 (Chicago, IL, United States) was used to analyze the data.

## Results

The participants placed in the HL and BFR training interventions were of similar age, mass, height, body mass index, and physical function upon entry into the study (*P* > 0.05; Table 1). There was 100% compliance in the 24 exercise sessions completed by both the HL and BFR training groups. Systolic and diastolic blood pressure values before and after the training did not change (time main effect *P* > 0.05 for both variables, η^2^ < 0.06; time x group interactions *P* > 0.05, η^2^ < 0.03). No adverse events occurred during the study.

Cross-sectional area of the quadriceps increased 6.5 ± 3.1% and 7.8 ± 8.2% in the HL and BFR training groups, respectively (Table 2). There was not a time × group interaction (*P* = 0.86, η^2^ = 0.01) or group main effect (*P* = 0.65, η^2^ < 0.01) but there was an overall main effect of time (*P* < 0.01, η^2^ = 0.66) as the growth in CSA was significant. A similar trend was evident in the CSA of the hamstrings as the HL and BFR training groups improved 5.3 ± 7.4% and 4.8 ± 5.9%, respectively. There was a main effect of time (*P* = 0.003, η^2^ = 0.38) and the time x group interaction (*P* = 0.62, η^2^ < 0.01) and group main effect (*P* = 0.86, η^2^ = 0.01) were not significant.

**Table 2:** Muscle strength and mass measurements before and after high-load (HL) and blood flow restricted (BFR) training presented as mean (standard deviation).

	HL			BFR		
		
	Pre	Post	% change	Pre	Post	% change
Knee extension						
10-RM (kg)^†^	39.0 (17.8)	60.5 (25.3)	59.9 (33.0)	36.6 (17.6)	47.1 (20.0)	35.8 (27.8)
MVC (Nm)^∗^	103.8 (36.1)	126.0 (42.5)	23.1 (13.4)	115.9 (38.5)	128.0 (45.4)	11.0 (21.9)
Quadriceps CSA (cm^2^)^∗^	44.7 (11.7)	47.7 (11.7)	6.5 (3.1)	45.4 (11.7)	48.9 (13.2)	7.8 (8.2)
**Knee flexion**						
10-RM (kg)^∗^	26.0 (10.3)	34.8 (8.0)	40.4 (28.3)	28.5 (10.2)	33.2 (12.6)	18.4 (22.5)
MVC (Nm)^∗^	60.7 (18.4)	68.9 (23.0)	13.7 (14.5)	63.4 (19.6)	69.5 (19.3)	11.9 (17.3)
Hamstrings CSA (cm^2^)^∗^	22.6 (7.4)	23.5 (7.1)	5.3 (7.4)	21.3 (6.0)	22.1 (6.2)	4.8 (5.9)


*^∗^Indicates significant main effect of timepoints for the overall sample (*P* < 0.05). ^†^Indicates significant time x group interaction (*P* < 0.05).*

The HL training group experienced a 60 ± 33% improvement in 10-RM in the KE exercise while the BFR training group had a 36 ± 28% increase (time x group interaction *P* = 0.02, η^2^ = 0.25; Table 2) however, there were no differences between the groups in the magnitude of strength gains in the KF 10-RM (time × group interaction *P* = 0.11, η^2^ = 0.13). Both groups experienced increases in KE MVC and KF MVC (time main effect *P* < 0.01 for both variables, η^2^ = 0.47 and 0.40, respectively; time x group interactions *P* > 0.05, η^2^ < 0.10 Table 2).

**FIGURE 1:**
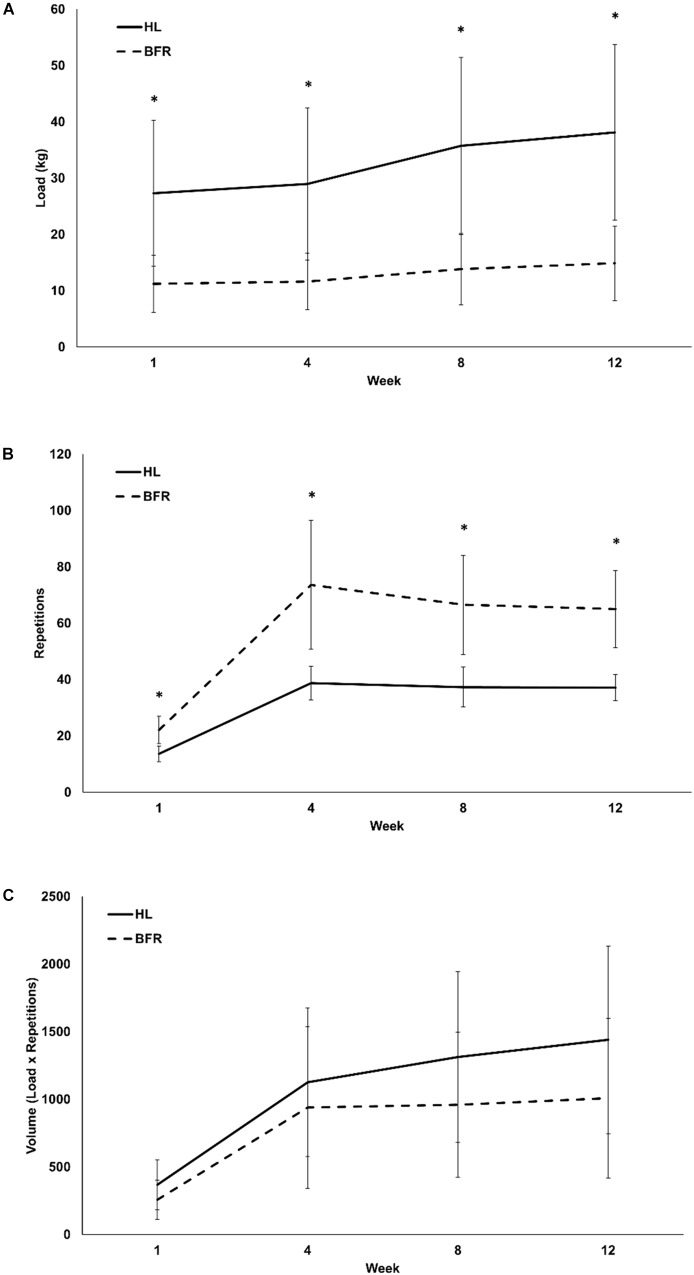
Monthly load **(A)**, repetitions **(B)**, and volume **(C)** of high-load (HL) and low-load blood flow restricted (BFR) knee extension resistance training. ^∗^ denotes significant difference between HL and BFR.

**FIGURE 2:**
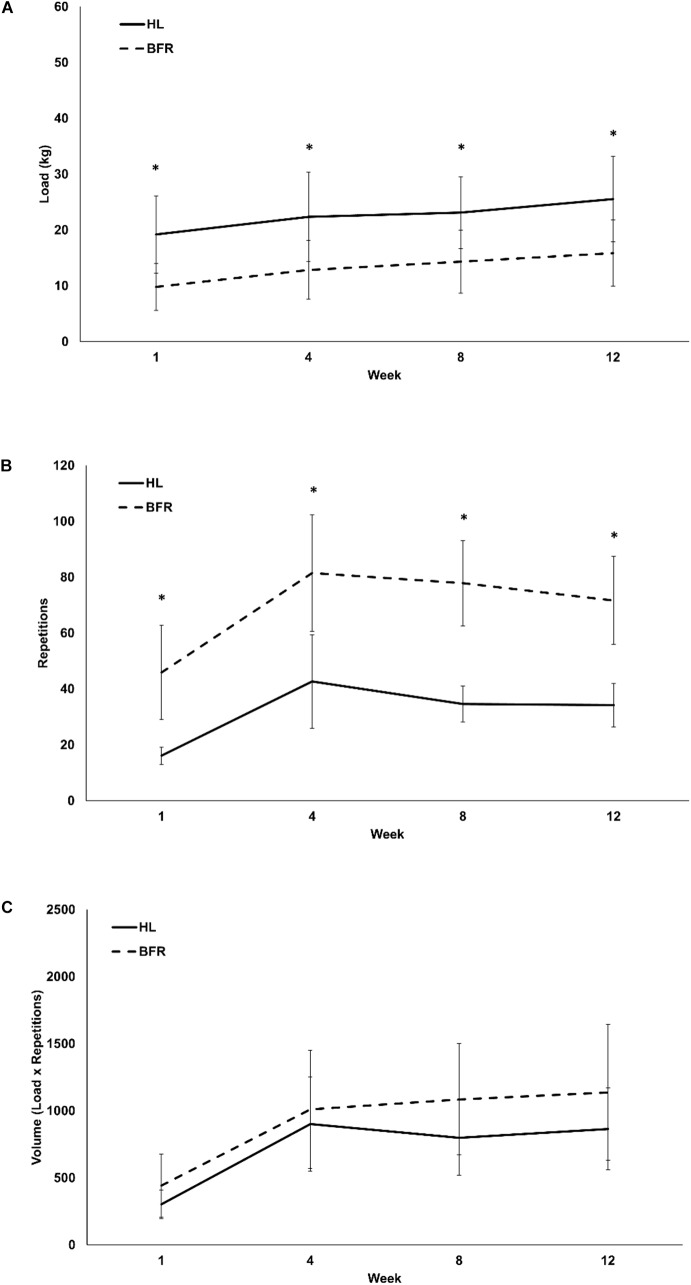
Monthly load **(A)**, repetitions **(B)**, and volume **(C)** of HL and low-load blood flow restricted (BFR) knee flexion resistance training. ^∗^ denotes significant difference between HL and BFR.

Both groups combined demonstrated significant progression in KE and KF loads, repetitions and volume throughout the 12-week training period (time main effect *P* < 0.01 for all variables; η^2^ > 0.53). There were significant group main effects for KE and KF loads (*P* < 0.01; η^2^ > 0.40) as the load was always higher in the HL group than the BFR group. KE and KF repetitions (*P* < 0.01; η^2^ > 0.70) in the BFR group was always higher than the HL group (Figure 1C, 2C). Despite this, there were no group differences in KE volume (*P* = 0.20; η^2^ = 0.08) and KF volume (*P* = 0.14; η^2^ = 0.11) (Figure 1, 2). There were significant time x group interactions in KE load (*P* < 0.01; η^2^ = 0.27) and KE repetitions only (*P* < 0.01; η^2^ = 0.29) (Figure 1A,B).

The KE load in the HL training group was always higher at weeks 1 through 12 than the BFR training group (*P* < 0.01; η^2^ = 0.47) while the BFR training group always performed more repetitions than the HL training group (Figure 1A,B). Overall, the HL group averaged an increase of 10.80 ± 7.11 kg from the beginning to the end of the study (0.90 ± 0.60 kg⋅week^-1^) in the KE exercise. This was significantly greater than the BFR group as they averaged 3.6 ± 2.6 kg (0.30 ± 0.21 kg⋅week^-1^); (*P* < 0.01; Table 3). However, these differences disappeared when they were expressed in relative terms as percent change (Table 3) as both groups combined averaged a 40.6 ± 29.5% and 192.5 ± 74.9% increase in KE load and repetitions, respectively. The KE load in the HL training group was lowest within the first four weeks of training when compared to week 8 and it continually increased up until week 12 (*P* < 0.01). The KE load in the BFR training group was also lowest within the first four weeks of training compared to weeks 8 and 12, however, there were no further increases in KE load from weeks 8 to 12 (*P* = 0.25) (Figure 1A).

**Table 3:** Absolute (kg) and relative (%) differences between load, repetitions and volume between weeks 1 and 12 in HL and blood flow restricted (BFR) training.

	HL		BFR	
		
	Absolute (kg)	Relative (%)	Absolute (kg)	Relative (%)
**Knee extension**				
Load (kg)	10.8 (7.1)^*^	46.1 (35.1)	3.6 (2.6)	34.6 (22.0)
Repetitions	23.5 (5.0)^*^	181.3 (63.7)	42.9 (14.0)	204.8 (87.5)
Volume (kg)	1072.0 (558.1)	195.6 (82.1)	750.7 (511.7)	190.3 (129.6)
**Knee flexion**				
Load (kg)^∗^	6.3 (1.7)	35.7 (13.7)^*^	6.0 (2.9)	67.4 (41.2)
Repetitions	18.1 (8.3)	120.7 (69.5)	25.7 (29.2)	78.7 (78.4)
Volume (kg)	561.1 (235.1)	305.7 (118.6)	694.6 (476.2)	312.5 (135.1)


*Data are presented as mean (standard deviation). ^∗^Indicates significant difference from BFR (*P* < 0.05).*

The BFR group completed more repetitions in the KE than the HL training group (3.6 ± 1.3 repetitions⋅week^-1^ vs. 2.20 ± 0.43 repetitions⋅week^-1^; *P* < 0.01; Figure 1B). The HL and BFR training groups significantly increased the repetitions performed from week 1 to weeks 4, 8, and 12 (*P* < 0.01), but the repetitions were constant from weeks 4 to 12 (*P* > 0.05). KE exercise volume (Figure 1C) increased over the 12 weeks for both groups combined (time main effect *P* < 0.01; η^2^ = 0.71) but there were no time x group interactions for KE volume (*P* = 0.44; η^2^ = 0.07). RPE in the KE exercise was similar between the HL and BFR training groups (*P* = 0.52; η^2^ = 0.04) but on average increased significantly from the week 1 (6.0 ± 2.0) to weeks 4, 8, and 12 (8.0 ± 2.0) (time main effect *P* < .01; η^2^ = 0.66).

Over the 12 weeks of training there were significant increases in KF load (*P* < 0.01; η^2^ = 0.69; Figure 2A), repetitions (*P* < 0.01; η^2^ = 0.53; Figure 2B), and volume (*P* < 0.01; η^2^ = 0.71; Figure 2C). The absolute change in KF load from the first to the twelfth week was not different between the training interventions (HL: 6.3 ± 1.7 kg or 0.53 ± 0.14 kg⋅week^-1^, BFR: 6.0 ± 2.9 kg, or 0.50 ± 0.50 kg⋅week^-1^, *P* = 0.77). However, when expressed as a percent of the first week of training, the BFR group experienced a 64.7 ± 41.2% increase in KF load while the HL group had an overall increase of 35.7 ± 13.7% (*P* = 0.03; Table 3). KF repetitions were not different between HL (18.1 ± 8.3 repetitions or 1.5 ± 0.69 repetitions⋅week^-1^) and BFR (25.7 ± 29.2 repetitions or 2.1 ± 2.4 repetitions⋅week^-1^) (*P* = 0.42; Table 3). RPE in the KF exercise was similar between the HL and BFR training groups (*P* = 0.17; η^2^ = 0.09) but on average increased significantly from the week 1 (6.0 ± 2.0) to weeks 4, 8, and 12 (8.2 ± 1.7) (time main effect *P* < .01; η^2^ = 0.75).

## Discussion

The findings from this study indicate that how exercise loads and repetitions are progressed likely have an impact on the muscular adaptations following resistance training in older adults. Increases in muscle mass and strength were evident in older adults at risk of mobility limitations following similar volumes of HL and BFR resistance training on the KE and KF muscle groups. However, the disparate strength gains in KE 10-RM in the HL training group may be due to multiple factors that could affect how researchers and clinicians implement and evaluate BFR exercise.

The similar gains in muscle mass following HL and BFR resistance training align with previous research on young adults (Laurentino et al., 2012; Martín-Hernández et al., 2013; Ellefsen et al., 2015) and older adults (Vechin et al., 2015). Even though no statistically significant differences were evident in strength gains in our study, we should consider that the percent change in KE 10-RM, KF 10-RM, and KE isometric MVC strength gains in HL training were approximately double than levels after BFR training. These diverse magnitudes of strength gains in the KE following BFR training have been reported in other studies in young adults (Martín-Hernández et al., 2013) and older adults (Karabulut et al., 2010; Vechin et al., 2015) and has been further described in a recent meta-analysis (Lixandrão et al., 2018). The comparable levels of KE muscle hypertrophy and disparate gains in KE strength imply that neural factors may play a key role. It also highlights the premise that preserving and gaining muscle mass in older adults may not be enough to impact physical function (Kim et al., 2012) and we must continue to optimize resistance training since it is the primary treatment for sarcopenia (Liguori et al., 2018).

One such component to further explore is the rate of change in KE exercise load during HL and BFR training. Despite having a lower rate of change in the number of repetitions performed when compared to BFR training, HL training had a much greater absolute change in load while volume in both training protocols remained constant. Our research quantifies the rate of progression in the KE exercise based on changes in load throughout the 12-week training duration of the study. The participants in the HL group experienced weekly increases in load of approximately 0.90 ± 0.60 kg⋅week^-1^ as they began with an average training load of 27 kg and progressed to 38 kg in the final week of training. The rate of progression in the HL group was three times greater than the BFR training group. Our previous publication using the same sample of participants demonstrated that most strength gains from HL and BFR exercise were obtained within the first 6-weeks of the resistance training program and only the HL group continued to have significant gains in the KE from weeks 6 to 12 (Cook et al., 2017). One of two situations may be possible based on the results of our research. First, it is reasonable to assume that the strength benefits from BFR resistance exercise are gained within the first few weeks of resistance training after which further improvements can only occur through HL resistance training. This aligns with the clinical application of utilizing BFR resistance exercise in patients undergoing bed rest and progressing them to HL resistance training (Loenneke et al., 2012a). The second situation may be that the progression of BFR KE exercise focuses more on loads rather than repetitions. For example, in our study the BFR group began training at an average load of 11 kg and progressed at a rate of 0.30 ± 0.21 kg⋅week^-1^ to a 15 kg load at the final week of training. If the BFR training group progressed at the same rate as the HL training group, their final load would be approximately 22 kg which would still be considered a low load of 46% 1-RM. Interestingly, when expressing these differences in load and repetitions in relative terms based on percent change, these differences disappear. Irrespective of how the changes in sets and repetitions are described, consideration of resistance exercise progression in older adults deserves future investigation.

It is well known that resistance exercise at high as well as low loads lead to enhanced strength in novice resistance trainers mainly due to improvements in motor learning and coordination (Rutherford and Jones, 1986; Kraemer and Ratamess, 2004). Further neuromuscular adaptations then arise from enhanced motor unit recruitment, rate coding and synchronization that may require loads of 80–85% of 1-RM to result in strength gains in advanced resistance trainers (Kraemer and Ratamess, 2004). In our study, the participants in the HL training group experienced significant increases in KE load throughout the entire 12-weeks of the study while the BFR training group had significant increases in load only within the first 4-weeks of the study. This difference in training progression may be an area for future studies to control for when implementing BFR resistance training protocols. It should also be considered that the 10-RM strength tests used loads most similar to those employed in the HL training program and as a result, 10-RM testing may have been more sensitive to the strength improvement of HL training than the BFR training that was performed at lighter loads. Researchers have suggested the use of isometric MVC strength as a neutral test to assess effectiveness of resistance training protocols (Buckner et al., 2017). As such, in the present study there were improvements in KE MVC following HL and BFR training (23 ± 13% and 11 ± 22%, respectively).

It is interesting that the KF muscle group did not have as robust adaptations following HL and BFR training as the KE muscle group exhibited, despite the reporting of similar RPE values. We suspect that this may be due partly to the differences in neuromuscular properties within the lower limb muscles as it has been suggested that extensor motor neurons are more plastic and adapt more readily to activity-based interventions (Kirk et al., 2018). Nevertheless, the KF muscle group plays an important role in the gait cycle and deserves more attention in older adults. There is limited data on the effects of BFR resistance exercise on KF strength and hamstring CSA. To our knowledge, only two studies have evaluated this muscle group and the BFR exercise was done during rehabilitation from knee surgery. Ohta et al. (2003) reported a milder atrophy and strength recovery after surgery when compared to the heavily impacted quadriceps that lost significant muscle mass and strength. Similarly, Tennent et al. (2017) reported a 77% and 39% increase in KE and KF muscle strength, respectively following 12 sessions of postoperative physical therapy. The magnitude of change in the KE and KF were more than twofold increases when compared to the conventional therapy offered to the control group (Tennent et al., 2017). Our study noted similar, significant improvements in KF strength after both training programs. While the absolute changes in load and repetition progression were not different, there were relative differences such that the BFR training program had a greater percent increase in training load throughout the 12-weeks of training. This provides evidence that perhaps the initial KF training load (30% of 1-RM) was too low in the BFR training group. Investigating the effects of BFR exercise on KF strength and CSA should be further explored and KF exercise protocols should be evaluated to consider the rates of progression and manipulation of loads and repetitions.

The strengths of this study include the high compliance rates and the comprehensive assessment of resistance training progression in older adults classified as being at risk of mobility limitations. Despite not actually possessing mobility limitations but having a lot of variability in strength levels, the participants in our study benefitted from HL and BFR resistance training by gaining muscle mass, and improving muscle strength. Unfortunately, we did not conduct tests of muscular endurance and power and therefore we cannot make conclusions to determine the effect of the training interventions on those variables. It is suspected that the faster rate of progression in repetitions performed by the BFR group may lead to superior improvements in muscular endurance due to the principle of specificity. Enhanced muscular power leads to gains in physical function (Reid et al., 2015) and should be evaluated following BFR exercise interventions.

## Conclusion

HL and BFR resistance training increase muscle mass and strength in the KE and KF muscle groups. HL training leads to more robust and favorable strength adaptations in the KE 10-RM and it may be attributed to the greater load and/or faster rate of progression of the load throughout the 12-week training period and the specificity of the testing modality. Future research should be aimed at optimizing BFR protocols for systematic load progression throughout the entire training period for maximal gains in strength.

## Ethics Statement

This study was carried out in accordance with the Declaration of Helsinki as recommended by the University of New Hampshire Institutional Review Board. All participants provided written informed consent.

## Author Contributions

SC conceived and designed the study, collected the data, performed the analysis, and wrote the manuscript. CC performed the analysis and wrote the manuscript.

## Conflict of Interest Statement

The authors declare that the research was conducted in the absence of any commercial or financial relationships that could be construed as a potential conflict of interest.

## Abbreviations

BFR: blood flow restriction
CSA: cross sectional area
HL: high-load
KE: knee extension
KF: knee flexion
RM: repetition maximum
